# Integrative analysis of TBI data reveals *Lgmn* as a key player in immune cell-mediated ferroptosis

**DOI:** 10.1186/s12864-023-09842-z

**Published:** 2023-12-06

**Authors:** Liyan Yan, Xiaonan Han, Mingkang Zhang, Yikun Fu, Fei Yang, Qian Li, Tian Cheng

**Affiliations:** 1 Department of Orthopedics, The First Affiliated Hospital of Zhengzhou University, Zhengzhou University, Zhengzhou, 450052 Henan China; 2 Department of Neurobiology, School of Basic Medical Sciences, Capital Medical University, Beijing, 100069 China; 3 Present Address: Department of Biochemistry and Molecular Biology, School of Basic Medical Sciences, Capital Medical University, Beijing, 100069 China

**Keywords:** Lgmn, WGCNA, Ferroptosis, TBI, GEO

## Abstract

**Background:**

Traumatic brain injury (TBI) is a central nervous system disease caused by external trauma, which has complex pathological and physiological mechanisms. The aim of this study was to explore the correlation between immune cell infiltration and ferroptosis post-TBI.

**Methods:**

This study utilized the GEO database to download TBI data and performed differentially expressed genes (DEGs) and ferroptosis-related differentially expressed genes (FRDEGs) analysis. DEGs were further analyzed for enrichment using the DAVID 6.8. Immunoinfiltration cell analysis was performed using the ssGSEA package and the Timer2.0 tool. The WGCNA analysis was then used to explore the gene modules in the data set associated with differential expression of immune cell infiltration and to identify the hub genes. The tidyverse package and corrplot package were used to calculate the correlations between hub genes and immune cell infiltration and ferroptosis-marker genes. The miRDB and TargetScan databases were used to predict complementary miRNAs for the Hub genes selected from the WGCNA analysis, and the DIANA-LncBasev3 tool was used to identify target lncRNAs for the miRNAs, constructing an mRNA-miRNA-lncRNA regulatory network.

**Results:**

A total of 320 DEGs and 21 FRDEGs were identified in GSE128543. GO and KEGG analyses showed that the DEGs after TBI were primarily associated with inflammation and immune response. Xcell and ssGSEA immune infiltration cell analysis showed significant infiltration of T cell CD4^+^ central memory, T cell CD4^+^ Th2, B cell memory, B cell naive, monocyte, macrophage, and myeloid dendritic cell activated. The WGCNA analysis identified two modules associated with differentially expressed immune cells and identified *Lgmn* as a hub gene associated with immune infiltrating cells. *Lgmn* showed significant correlation with immune cells and ferroptosis-marker genes, including *Gpx4*, *Hspb1*, *Nfe2l2*, *Ptgs2*, *Fth1*, and *Tfrc*. Finally, an mRNA-miRNA-lncRNA regulatory network was constructed using *Lgmn*.

**Conclusion:**

Our results indicate that there is a certain correlation between ferroptosis and immune infiltrating cells in brain tissue after TBI, and that *Lgmn* plays an important role in this process.

## Introduction

Traumatic Brain Injury (TBI) refers to damage to the brain tissue caused by violent forces such as blows, vibrations, or impacts to the head. In China, the annual incidence rate of TBI is 55.4–64.1 cases per 100,000 people, which translates to approximately 770,060–890 new cases each year [1, 2]. It is estimated that TBI-related mortality accounts for 30–40% of all injury-related deaths, and TBI is projected to become the fourth leading cause of disability by 2030 [3]. TBI is primarily classified into primary and secondary injuries. Primary injury refers to the direct physical destruction of brain tissue caused by external forces, while secondary injury refers to a series of pathological reactions resulting from primary injury, including disruption of the blood-brain barrier (BBB), cerebral edema, infiltration and activation of peripheral immune cells, and so on, lasting for several months to years [4].

Iron-dependent regulated necroptosis, known as ferroptosis, is caused by extensive membrane damage mediated by lipid peroxidation [5, 6]. Ferroptosis can rapidly spread between cells through the release of oxidized lipids via extracellular vesicles, without depending on membrane rupture [7, 8]. Mitochondria are the metabolic center and major source of reactive oxygen species (ROS) in most mammalian cells. Studies have shown that mitochondrial-mediated ROS generation and oxidative stress-induced lipid peroxidation can induce ferroptosis [9–11]. At the ultrastructural level, ferroptotic cells typically exhibit mitochondrial abnormalities, such as condensation (early time points) or swelling (late time points), increased membrane density, decreased or missing cristae, and outer membrane rupture [6, 12]. Ferroptosis plays a crucial role in the pathological processes of various central nervous system diseases [13, 14]. After TBI, oxidative stress leads to the production of a large amount of ROS that attacks phospholipid membranes rich in polyunsaturated fatty acids (PUFAs), resulting in lipid peroxidation damage to the cell membrane [15].

In addition to neuronal ferroptosis, TBI can also induce immune cell infiltration and inflammation. After TBI, the BBB is disrupted, leading to the release of cellular contents from dead cells (known as damage associated molecular patterns, DAMPs), which induces various immune cells such as T cells, B cells, macrophages, etc., to enter the damaged brain area and release multiple inflammatory mediators, resulting in neuronal death and brain tissue inflammation [16]. Therefore, immune cell infiltration is closely related to the pathological processes of TBI.

Although both ferroptosis and immune cell infiltration play an important role in the pathological process of TBI, their relationship between them has not been thoroughly investigated. In this study, we obtained relevant data on TBI from public databases and used the Weighted correlation network analysis (WGCNA) method to analyze ferroptosis and immune cell infiltration. With this approach, we were able to identify genes associated with these two biological processes and construct a gene co-expression network to reveal their relationship. Our study suggests that there was a close relationship between these two biological processes. Specifically, we identified several important genes that may mediate the interaction between ferroptosis and immune cell infiltration.

## Materials and methods

### Microarray dataset collection and data process

We searched the GEO database using the following search terms: ‘TBI’, ‘Expression profiling by array’, and ‘Mus musculus’. The search strategy was defined as follows: (Traumatic brain injury) AND “Mus musculus” [porgn:__txid10090] Filters: Expression profiling by array. Ultimately, we selected GSE128543 as the training set. The GSE128543 database comprised data from seven TBI mice and seven Sham group mice. The samples were analyzed using the GPL20775 array, which contains a total of 73,070 DNA probes in total. We annotated the ID_REF based on GPL20775 annotations, and calculated the average expression values of multiple probes when multiple probes correspond to a gene.​.

### Screening differentially expressed genes and ferroptosis-related differentially expressed genes

We used the network analysis tool (https://www.networkanalyst.ca/) to analyze the expression of 23,423 genes in GSE128543. The standard setting for identifying Differentially Expressed Genes (DEGs) were adj.*p*.value < 0.05 and |logFC|>1. FerrDb (http://www.zhounan.org/ferrdb/current/) is a database of genes related to ferroptosis that have been experimentally verified. A total of 484 ferroptosis-related genes were extracted from FerrDb. DEGs and ferroptosis-related genes were overlapped using the Venn tool (https://bioinformatics.psb.ugent.be/webtools/Venn/) and the same genes were defined as Ferroptosis-Related Differentially Expressed Genes (FRDEGs). The expressions for the FRDEGs were visually analyzed, and a heatmap was constructed using the pheatmap package of R. Additionally, a volcano plot was constructed using the ggplot2 package.

### Functional enrichment analysis

To reveal the functional characteristics and interrelationships of DEGs involved in biological processes and pathways after TBI, we used the DAVID 6.8 to perform Gene Ontology (GO) and Kyoto Encyclopedia of Genes and Genomes (KEGG) enrichment analyses [17, 18]. The GO analysis included three categories: Biological Process (BP), Cellular Component (CC), and Molecular Function (MF). The significance threshold for presenting the results was set at FDR < 0.05.

### Immune cells infiltrating

Single-sample Gene Set Enrichment Analysis (ssGSEA) is an immunoassay that scores individual samples based on immune-associated gene sets to assess the expression levels of different immune infiltrate-associated cells in the sample. Xcell is similar to ssGSEA in that it is also an immune cell analysis tool used to predict the contents of various immune cells in a sample by analyzing the input gene expression data.

To perform the ssGSEA analysis on the GSE128531 dataset, we used the GSVA package in R to analyze 23,423 genes, and quantified the results for 28 immune cell species [19]. The Xcell analysis was performed using the Timer2.0 tool (http://timer.cistrome.org/), quantifying the results for 36 immune cells, and presented the results using the pheatmap package [20]. We performed immune cell infiltration analysis using ssGSEA and Xcell, and obtained the overlapping results from both methods.

### Construction of weighted gene co-expression network analysis

The WGCNA package and flashclust package in R were used to perform Weighted Gene Co-Expression Network Analysis (WGCNA) analysis [21]. Firstly, we calculated the standard deviations of all genes and selected the top 5000 genes for further analysis. The soft threshold β was determined to be around 0.9 by calculating the soft threshold, and a scale-free network and Topological Overlap Matrix (TOM) were constructed to classify genes with highly correlated expression into the same color module. Similar modules were merged according to the TOM and the corresponding dissimilarity (1-TOM), and the TOM value was set at 0.25.

Next, we screened the modules with the highest correlation between different modules and immune infiltrating cells. Then, we calculated the Hub-gene for further analysis.

### Hub-gene correlation analysis

We used the tidyverse package to calculate the correlation between Hub-gene and immune cells, then visualized the data. We also used the corrplot package to analyze the correlation between Hub-gene and ferroptosis marker-gene, and drew the correlation matrix diagram with the results.

### Construction of ceRNA network

In this study, we constructed a ceRNA regulatory network consisting of mRNA-miRNA-lncRNA interactions. MiRNAs regulate gene expression levels by complementary pairing with mRNAs, leading to translational repression or degradation of mRNAs. LncRNAs are also involved in gene expression regulation, affecting gene expression in a variety of ways, including regulation of transcription factor activity, regulation of RNA splicing, and inhibition of miRNA activity. We used the miRDB (https://mirdb.org/) and TargetScan (https://www.targetscan.org) databases to predict the complementary miRNAs of Hub-genes, which were screened by WGCNA analysis [22, 23]. The prediction results were obtained by overlapping data from both databases. We then used the DIANA-LncBasev3 tool (https://diana.e-ce.uth.gr/lncbasev3/expression) to target lncRNAs for miRNAs [24]. Finally, we constructed and visualized the mRNA-miRNA-lncRNA regulatory network using Cytoscape.

### Statistical analysis

The data were analyzed using SPSS21.0 software and presented as mean ± SD. The independent samples *t*-test was employed to determine the difference between two groups for data that followed a normal distribution and met the assumption of homogeneity of variance. If the data did not meet the assumptions of normal distribution and homogeneity of variance, the Mann-Whitney U nonparametric test was used. A p-value of < 0.05 was considered statistically significant.

## Results

### DEGs and FRDEGs expression

To identify the genes involved in TBI and understand their role and function in the pathophysiology of the disease, we downloaded the GSE128543 dataset from the GEO database. This data set includes a total of 14 mice with TBI induced by cortical impact, including 7 sham mice (anesthetized but not subjected to craniotomy) and 7 TBI mice (underwent craniotomy and cortical impact injury in the left parietal cortex). All mice were sacrificed at 48 h of injury for microarray analysis.

**Fig. 1:**
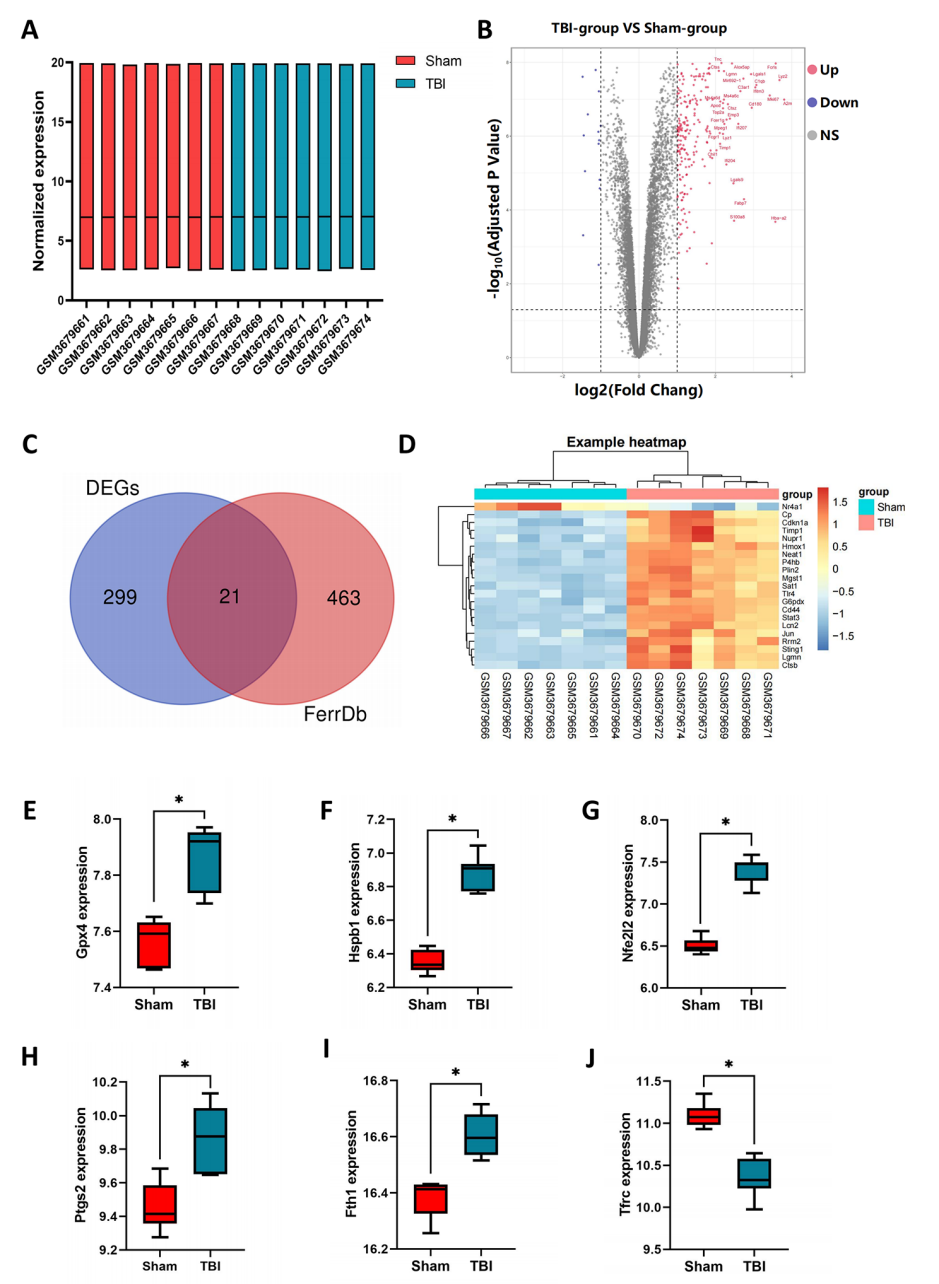
Identification of DEGs and FRDEGs. **(A)** Consistency of data from 14 samples. **(B)** Volcano plot showing the DEGs in the GEO dataset. Up-regulated genes are shown in red, while down-regulated genes are shown in blue. Up-regulated genes are shown in red, while down-regulated genes are shown in blue. The black line inside the box represents the median. **(C)** Venn diagram showing the overlapping genes between the DEGs and the FerrDb database. **(D)** Heatmap displaying the expression levels of the 21 FRDEGs. **(E-J)** The expression of ferroptosis marker-genes were altered in TBI mice in the GSE128543 dataset. The independent samples *t*-test. The black line inside the box represents the median. *P＜0.05. Each group contained 7 mice

The dataset contained a total of 23,423 genes, and the box plots showed robust data consistency (Fig. 1A). Network analysis was performed to analyze the mice of sham group and TBI group, and 320 DEGs were obtained (Fig. 1B). Compared with the Sham group, there were 315 upregulated genes and 15 downregulated genes (adj.*p*-value < 0.05 and |logFC|>1) in the TBI group. The ferroptosis-driver, ferroptosis-suppressor, and ferroptosis-marker genes were downloaded from the FerrDb database, and 484 ferroptosis-related genes were obtained after removing duplicate entries. The DEGs and 484 ferroptosis-related genes were overlapped using Venn diagram (Fig. 1C). Finally, 21 FRDEGs were obtained and displayed using a heatmap (Fig. 1D). In the GSE128543 dataset, there were a total of 6 ferroptosis-marker genes with statistical differences, among which *Gpx4*, *Hspb1*, *Nfe2l2*, *Ptgs2*, and *Fth1* were upregulated in the TBI group, while *Tfrc* was downregulated in the *TBI* group (Fig. 1E-J).

### Enrichment analysis

**Fig. 2:**
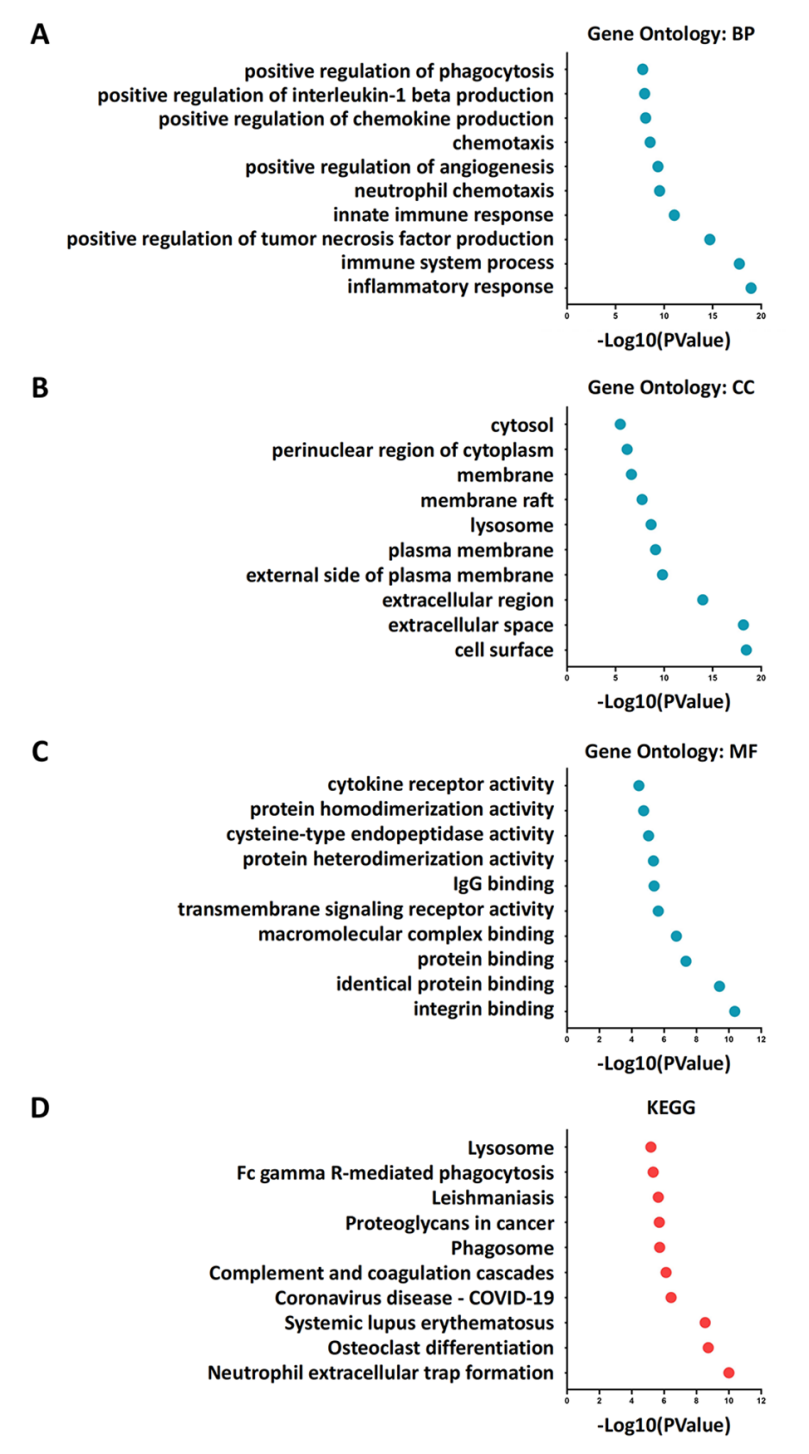
Enrichment analysis. **(A)** The top 10 GO biological process pathways. **(B)** The top 10 GO cellular component pathways. **(C)** The top 10 GO molecular function pathways. **(D)** The top 10 KEGG pathways

The enrichment analysis of 320 DEGs was performed using the DAVID 6.8. A total of 368 biological processes, 88 molecular functions, 79 cellular components, and 67 signaling pathways were enriched. Based on the GO enrichment results, the DEGs were found to be associated with several biological processes, including inflammatory response, immune system process, innate immune response, neutrophil chemotaxis, and chemotaxis (Fig. 2A). Additionally, the analysis of cellular components showed that these DEGs were expressed in various cellular components, such as the cell surface, extracellular space, extracellular region, external side of the plasma membrane, and plasma membrane, etc. (Fig. 2B). Molecular functional analysis showed that these DEGs were mainly related to integrin binding, identical protein binding, protein binding, macromolecular complex binding, and transmembrane signaling receptor activity, etc. (Fig. 2C). Furthermore, KEGG pathway analysis of DEGs using David showed that these DEGs were mainly associated with neutrophil extracellular trap formation, osteoclast differentiation, systemic lupus erythematosus, complement and coagulation cascades, and phagosome, etc. (Fig. 2D). Overall, the results of the enrichment analysis were mostly related to immune and inflammatory function. To gain a deeper understanding of the function and role of these genes, we subsequently performed immune infiltration cell analysis to identify the different types of immune cells involved in inflammation and immune response during TBI.

### Immune infiltrating analysis

**Fig. 3:**
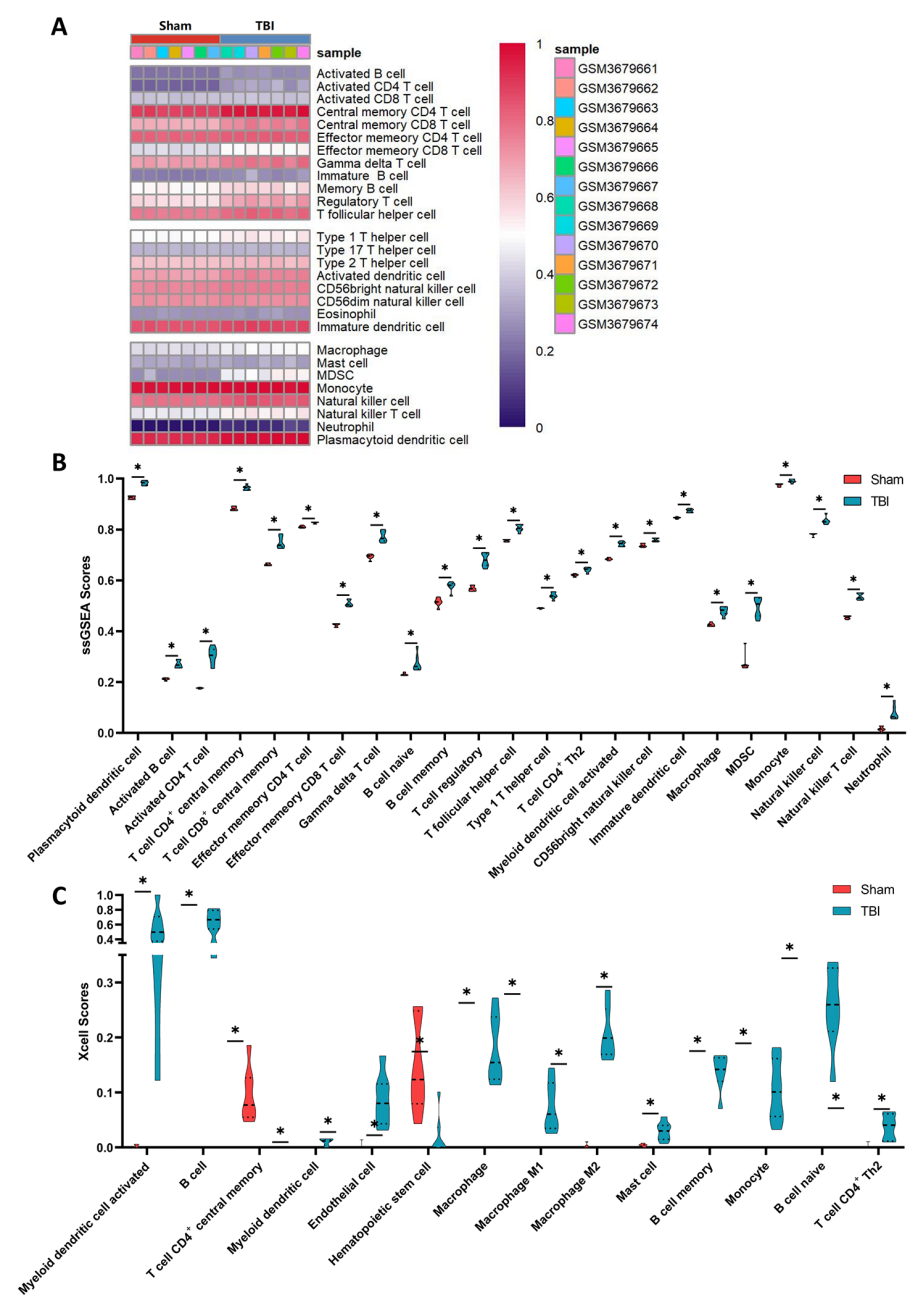
Evaluation and correlation analysis of immune cell infiltration. **(A)** The score of 36 types of immune infiltrating cells by ssGSEA. **(B)** The 22 immune cells with differences in ssGSEA scores. **(C)** The 17 immune cells with differences in Xcell scores. The significance represented by “*” is *P* < 0.05

Xcell and ssGSEA can be used to infer the relative proportions of different types of immune cells in a sample, using different algorithms for the calculation. We used both methods simultaneously for immune infiltrate cell analysis and validated the results through mutual corroboration to reduce errors and improve the reliability of the result. The ssGSEA results were quantified (Fig. 3A). A total of 22 differentially expressed immune cells were identified by ssGSEA (Fig. 3B), and 17 differentially expressed immune cells were identified by Xcell (Fig. 3C). Among the results of Xcell and ssGSEA, a total of 9 immune cells showed generally consistent results: T cell CD4^+^ central memory, T cell CD4^+^ Th2, B cell memory, B cell naive, monocyte, macrophage, myeloid dendritic cell activated.

### Construction of WGCNA network and identification of immune-related modules and hub genes

**Fig. 4:**
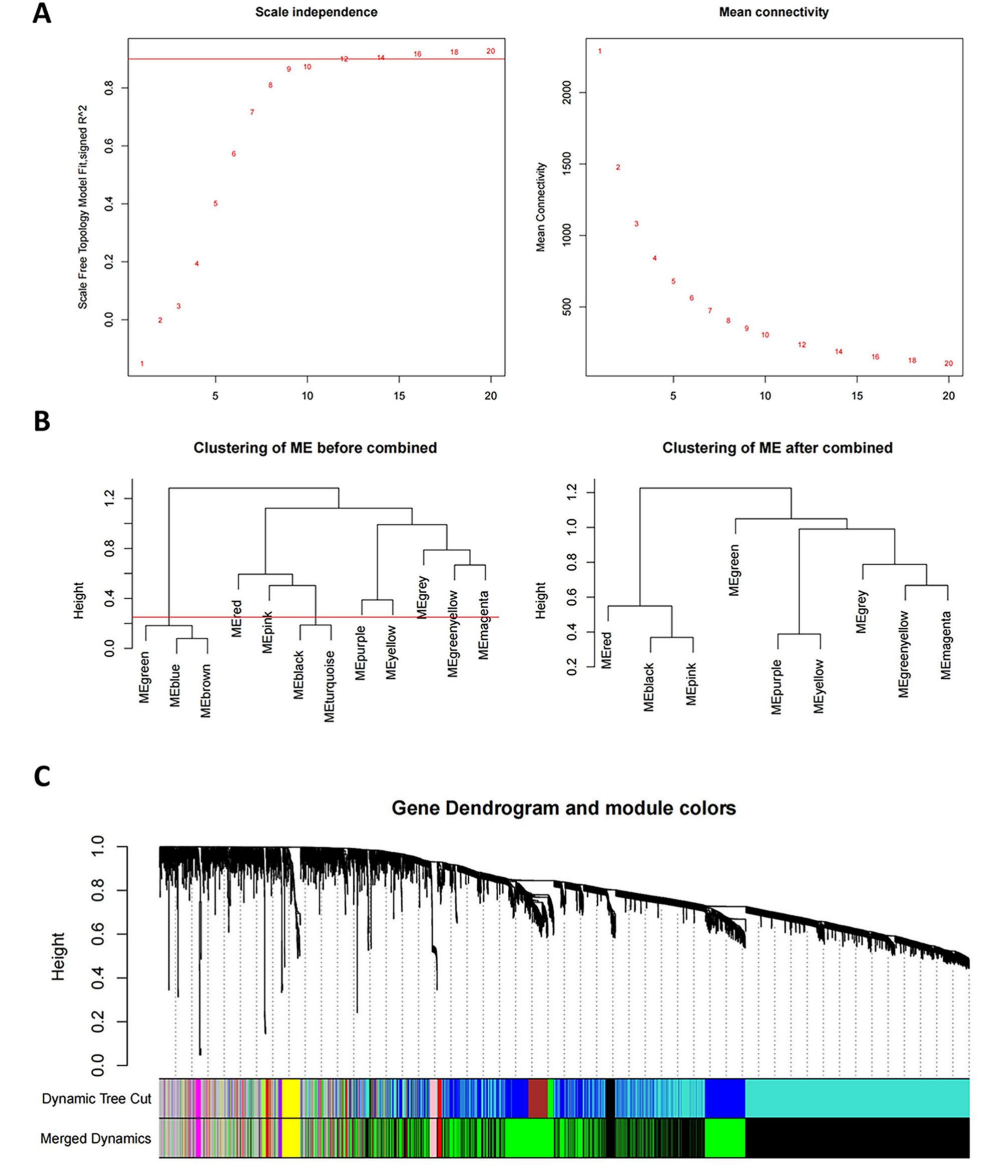
WGCNA network construction. **(A)** Network topology analysis for different soft thresholds. In the case of R^2^ > 0.9, 12 was chosen as the most suitable power value. **(B)** Merging of modules based on TOM. **(C)** Clustering dendrogram of genes based on topological overlap of dissimilarity and assigned module colors

**Fig. 5:**
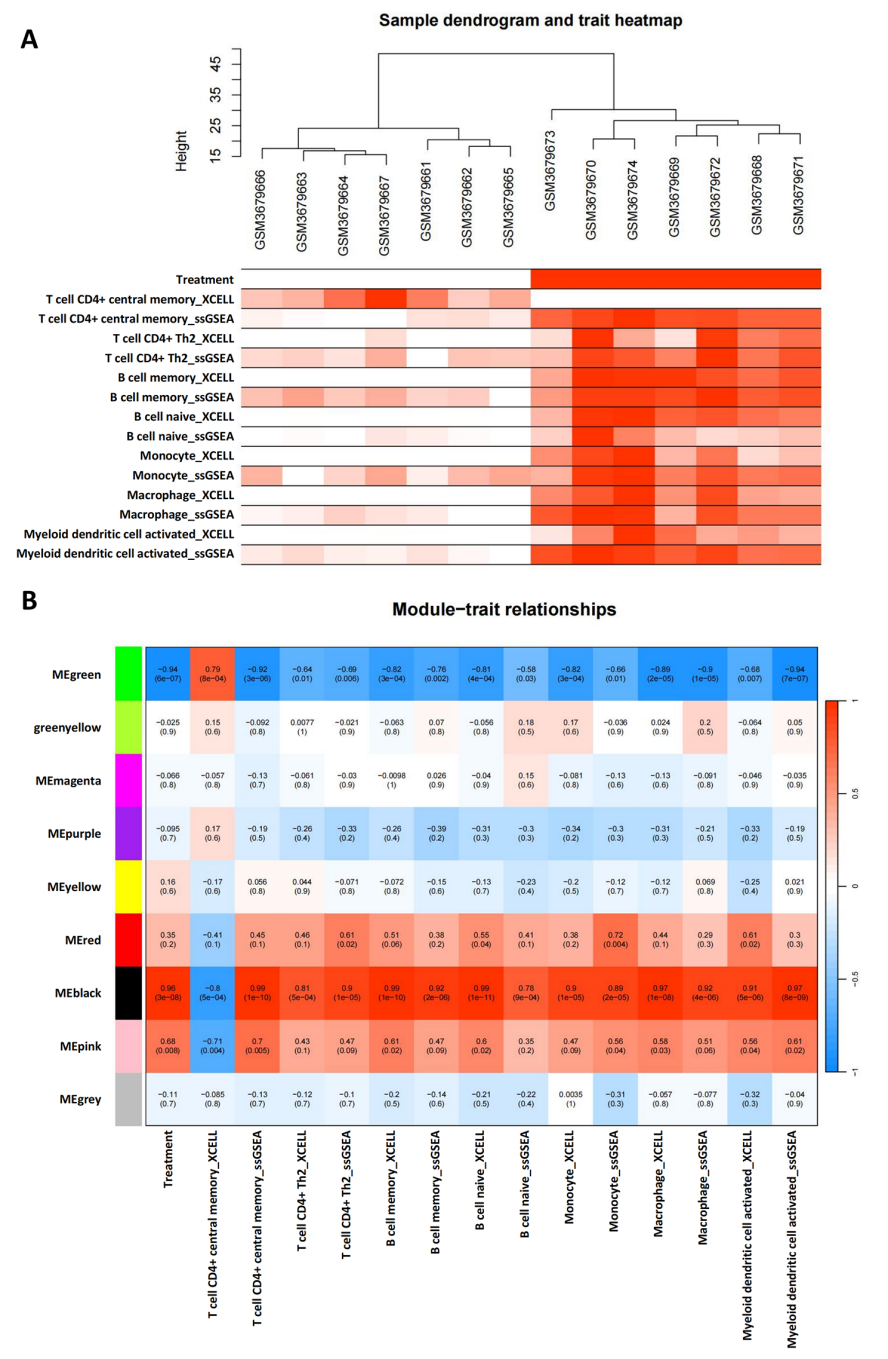
Module correlation analysis. **(A)** Cluster dendrogram based on sample feature information. Dark colors indicate high correlation, while light colors represent low expression levels. **(B)** Correlation analysis of different color modules with sample feature information

In order to gain a deeper understanding of the roles and interactions of different types of immune cells in TBI, we used WGCNA analysis to cluster genes into different gene modules. Then, we performed an association analysis between these gene modules and the results of immune infiltrating cells, and identified key modules associated with different immune cell types. The GSE128543 was used to construct WGCNA network, when R^2^ reaches 0.9, the soft threshold power β is 12, and the average connectivity is stable, indicating that the network connection is excellent (Fig. 4A). We used the WGCNA package to construct the co-expression module, and the gene module was obtained by dynamic mixed cutting. After removing the similar modules (TOM = 0.25), 9 modules (Fig. 4B, C) were generated: MEgreen, greenyellow, Memagenta, Mepurple, Meyellow, Mered, Meblack, Mepink, and Megrey. Correlation analysis was performed to calculate the correlation between the gene modules and immune information (Fig. 5A, B). The results showed that the Black module and Green module had the strongest correlation with immune infiltrating cells. We extracted the Hub genes of the Black and Green modules, which were *Lgmn* and *Ntm*, respectively. *Lgmn* is a FRDEG, so we chose *Lgmn* for further analysis.

**Fig. 6:**
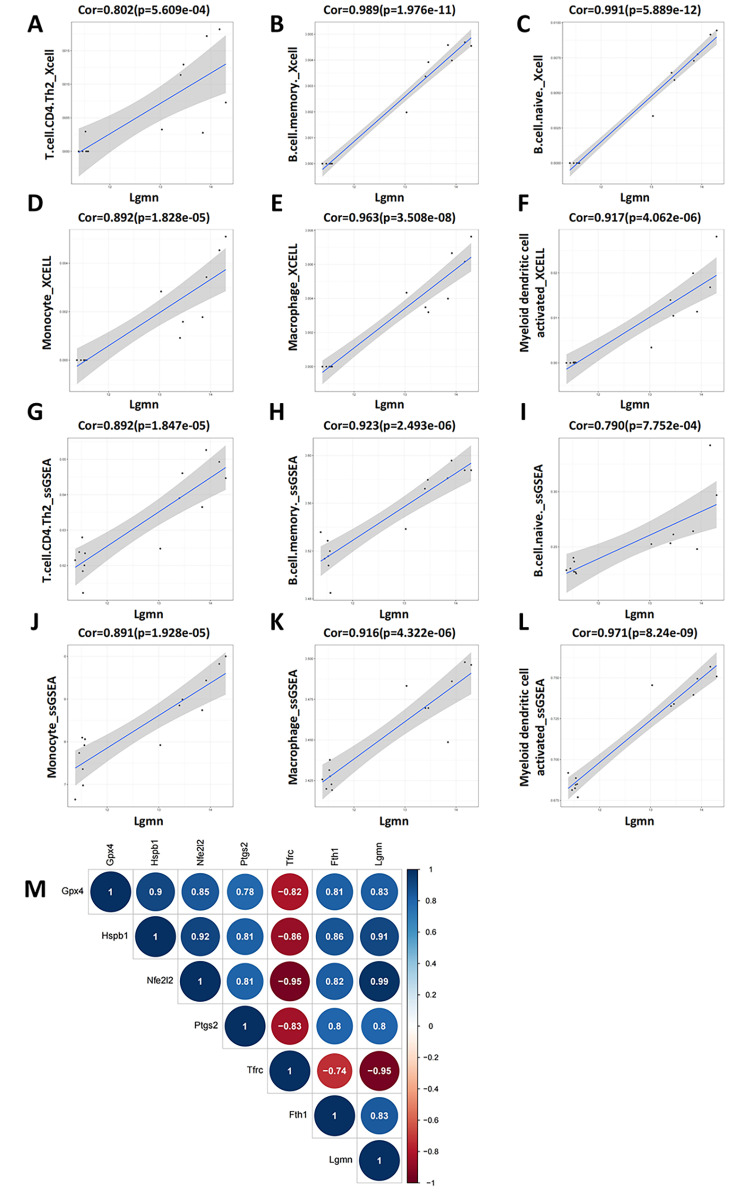
Correlation analysis of hub genes. **(A-F)** Correlation between hub genes and 6 types of immune cells in Xcell. **(G-L)** Correlation between hub genes and 6 types of immune cells in ssGSEA. **(M)** Correlation between hub genes and ferroptosis marker genes

In the correlation analysis between modules and immune information, the correlation between modules and T cell CD4^+^ central memory was different. We excluded T cell CD4 + central memory and used the tidyverse package to analyze the correlation between *Lgmn* and the remaining six kinds of immune cells. The results showed that *Lgmn* was positively correlated with six kinds of immune infiltrating cells (Fig. 6A-L). At the same time, using the corrplot package to analyze the correlation between *Lgmn* and ferroptosis marker genes, it was found that *Lgmn* were positively correlated with *Gpx4*, *Hspb1*, *Nfe2l2*, *Ptgs2*, and *Fth1*, and negatively correlated with *Tfrc* (Fig. 6M). *Lgmn* encodes Legumain protein, also known as asparagine endopeptidase (AEP), which mainly exists in lysosomes and participates in the processing of various proteins, and plays an important role in immune reactions [25]. Recent study has shown that *Lgmn* may mediate renal tubular ferroptosis through the glutathione peroxidase 4 (GPX4) autophagy mechanism, leading to acute kidney injury (AKI) [26]. These results indicate that *Lgmn* is closely related to ferroptosis.

### Construction of ceRNA network

In order to identify the potential regulatory mechanisms of the Hub gene *Lgmn* and its related targets, and to comprehensively elucidate the function and role of *Lgmn* in ferroptosis and immune cells, we have constructed an mRNA-miRNA-lncRNA interaction network to further investigate its function.

**Fig. 7:**
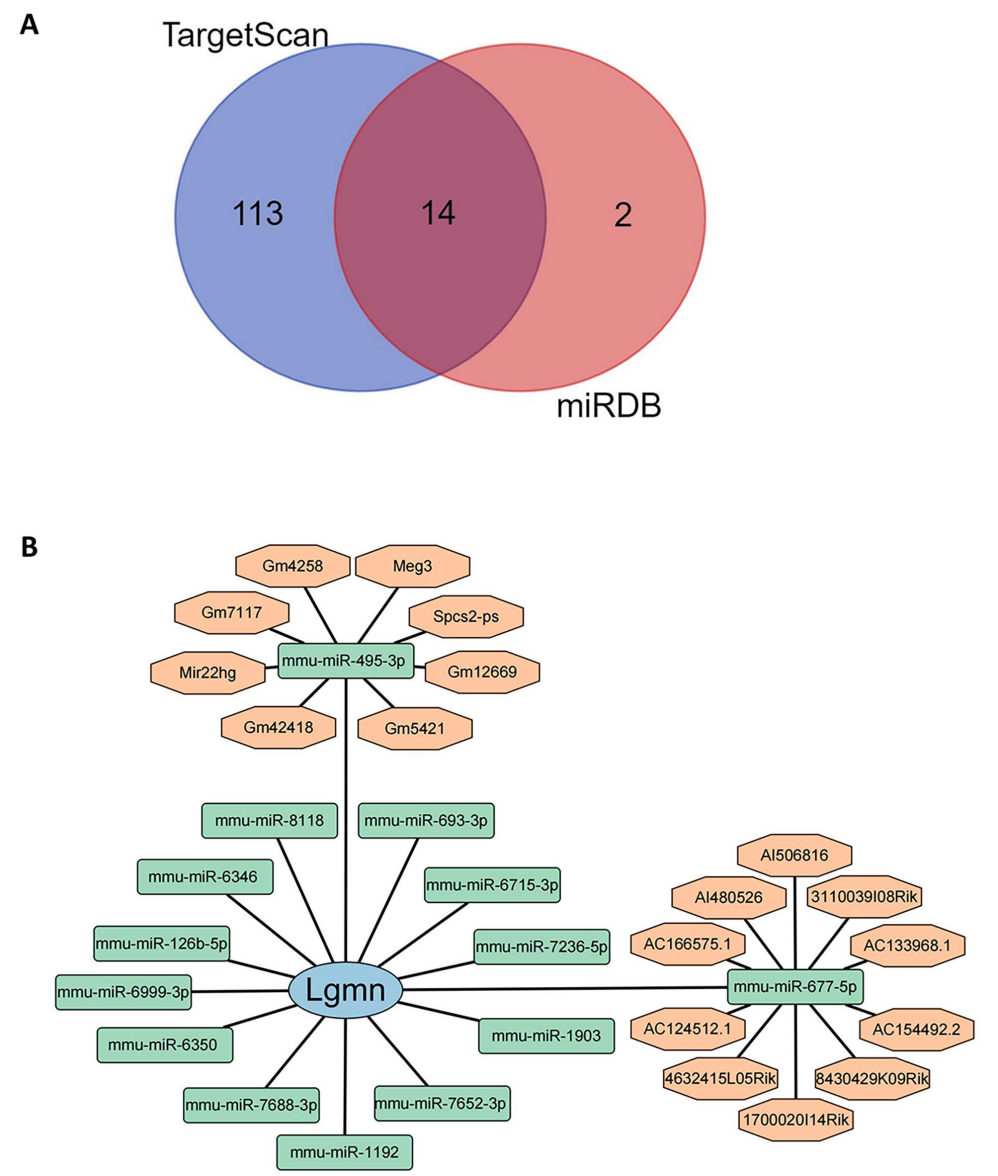
Construction of ceRNA network based on Hub Gene. **(A)** Identification of miRNAs that can bind to the hub gene by overlapping miRNA target predictions from miRDB and TargetScan. **(B)** Construction of the mRNA-miRNA-lncRNA interaction network based on the identified miRNAs and their target mRNAs and lncRNAs. The blue shapes represent mRNA, the green shapes represent miRNA, and the orange shapes represent lncRNA. Solid lines indicate a correlation

The Hub gene *Lgmn* was predicted using the miRDB and TargetScan databases, resulting in a total of 156 miRNAs. Among them, 14 miRNAs were found in both databases (Fig. 7A). Corresponding lncRNAs were further predicted through the DIANA-LncBasev3 database, resulting in 18 lncRNAs (Fig. 7B). Finally, the results were imported into Cytoscape to construct an mRNA-miRNA-lncRNA interaction network.

## Discussion

TBI refers to the damage caused to the brain structure and function by external forces, and is a serious public health problem characterized by high incidence, high disability, high mortality, and high economic burden [27]. TBI can cause neuronal injury and death, and lead to various neurological dysfunctions, such as motor and cognitive impairments, dementia, as well as psychiatric disorders such as anxiety and depression [28]. The pathophysiology of TBI is a complex and dynamic process involving both primary and secondary injuries. Primary injury is the direct mechanical damage caused by the force, while secondary injury begins at the time of injury and develops through the signaling by multiple interconnected molecular pathways. These pathways include inflammatory response, excitotoxicity, oxidative stress, and mitochondrial dysfunction [29]. Therefore, a comprehensive understanding of the pathophysiology of TBI is importance for the development of more effective treatments and preventive measures.

PUFAs are important components of cell membrane phospholipids and are involved in regulating various biological functions, including inflammation, immunity, synaptic plasticity, and cell growth [30, 31]. TBI causes oxidative stress, leading to the production of large amounts of ROS that attack the phospholipid membrane and cause lipid peroxidation damage [32]. Because the central nervous system is rich in PUFAs, it is susceptible to lipid peroxidation damage [33]. In 2012, Dixon first proposed the concept of iron-dependent cell death, known as ferroptosis [6]. In 2018, the Cell Death Nomenclature Committee proposed that ferroptosis be defined as a form of regulated cell death caused by oxidative stress in the cellular microenvironment and regulated by GPX4. The main causes of ferroptosis are inhibition of the system Xc- and GPX4, disruption of iron balance, and lipid peroxidation. Ultimately, the decline in cellular antioxidant capacity and accumulation of intracellular ROS lead to oxidative cell death [34, 35]. Glutathione (GSH) is an important intracellular antioxidant formed by glutamate, cysteine, and glycine under the action of gamma-glutamylcysteine synthetase and glutathione synthetase [30]. GPX4, a selenoprotein with a selenocysteine and seven cysteines in its active site, is the only GPX that can eliminate lipid peroxidation in biological membranes and inhibit cell death caused by iron [36–38]. Under the action of GPX4, GSH is converted to oxidized glutathione, while lipid hydroperoxides (L-OH) are reduced to the corresponding alcohols (L-OH), which can inhibit the production of lipid ROS and protect cells from oxidative stress damage [30]. Fe^2+^ binds to small molecules, including GSH, and is stored in cells [39]. Fe (II) is maintained in cells in the form of a labile iron pool, which binds to small molecules including GSH. Depletion of GSH not only inactivates GPX4, but also mobilizes Fe (II) to undergo Fenton chemistry, promoting the production of lipid peroxides and eventually leading to ferroptosis [39, 40]. Treatment with the ferroptosis inhibitor Lipostatin-1 in TBI mice resulted in reduced neuronal damage and degeneration, alleviation of brain edema, microglial activation, and neuroinflammation, and better protection of the BBB integrity [41].

In this study, we downloaded a transcriptomic dataset GSE128543 from the TBI-related GEO database, and identified the DEGs by differential analysis. To further investigate the role of these DEGs in ferroptosis, we compared them with the FerrDb database, and ultimately identified 21 FRDEGs that are closely related to iron metabolism regulation. In this data set, there are 6 genes (*Gpx4*, *Hspb1*, *Nfe2l2*, *Ptgs2*, *Fth1*, *Tfrc*) that have been confirmed to be ferroptosis-marker genes. *Gpx4* encodes GPX4, an important regulatory factor in the process of ferroptosis [38]. *Hspb1* encodes heat shock protein B1, which can reduce ferroptosis by lowering oxidative stress levels [42]. *Nfe2l2* encodes transcription factor Nrf2, which regulates the oxidative stress response [43]. *Ptgs2* encodes cyclooxygenase 2, which is involved in apoptosis and necrosis in ferroptosis [44]. The upregulation of these genes can effectively reduce the damage caused by ferroptosis. The upregulation of *Fth1* and the downregulation of *Tfrc* suggest a reduction in the ability of the cell to uptake iron, which is also a protective measure against iron overload. This suggests that iron ions accumulate in cells, as the protein encoded by the *Fth1* gene is an important regulator of iron metabolism [45], while the protein encoded by the *Tfrc* gene is the main channel for iron ion entry into cells [46]. In summary, although we were unable to determine the occurrence of ferroptosis in this dataset based solely on changes in the expression of these genes, these results still support the possibility of this hypothesis.

In addition, we performed GO and KEGG analyses on the DEGs. The enrichment analysis results revealed that the regulation of the immune response after TBI is related to multiple biological processes and molecular functions, and is associated with the occurrence a variety of diseases.

TBI is a complex neurological injury whose pathophysiology is still poorly understood. Immune cells play a crucial role in the occurrence and development of TBI. Changes in immune cells not only reflect the immune-inflammatory response after injury, but may also have an impact on the recovery and treatment of TBI [47]. Th1 and Th17 cells in the helper T cell subset can exacerbate inflammatory reactions and initiate immune responses, while Th2 cells can alleviate inflammation and exert protective effects after injury. Tanusree Sen observed an increase in Th2 cell infiltration, anti-inflammatory cytokines IL-4 and IL-10, and anti-inflammatory M2 microglial cell expression, as well as a decrease in white matter injury in brain tissue after treatment with GSK2656157 for TBI [48]. Naive B cells refer to B cells that have not yet been stimulated by a specific antigen and are in a resting state. Antigens can induce activation of naive B cells and further differentiation into plasma cells or memory B cells that secrete antibodies by binding to the B cell receptor [49]. Local application of naive B cells can promote wound healing in the skin, effectively promote tissue regeneration, and inhibit inflammatory reactions [50]. In vitro injection of naive B cells can significantly alleviate learning and memory impairments after central nervous system injury, reduce brain tissue loss, and exert neuroprotective effects [51]. In TBI, a large number of cells necrosis and release their cell contents, recruiting monocytes to enter the injury tissue from the blood into the injury tissue and activating resident microglial cells. Monocytes enter brain tissue through the bloodstream and then differentiate and mature into macrophages/microglial cells. Microglial cells are resident immune cells in the central nervous system, mainly responsible for phagocytosing and digesting microorganisms, dead cells, and other foreign substances in the body, and responding rapidly to inflammatory events in the nervous system [52]. After brain injury, monocytes infiltrate the injured area and peak at around 3–5 days after injury [53]. Monocytes recruit various inflammatory cells to the injury site through the secretion of CCL2 [54, 55], such as astrocytes and oligodendrocytes, which fill the damaged tissue and repair the injured brain, but have adverse effects on neuronal regeneration and functional recovery [56]. Dendritic cells are lacking in the central nervous system under steady-state conditions. However, a large number of dendritic cells accumulate in the central nervous system during central nervous system injury, recruiting and presenting antigens to antigen-specific T cells [57, 58].

WGCNA is a clustering, modularization, and hub gene identification method used for gene expression data. It enables comprehensive analysis of large-scale gene expression data by clustering expression profiles into multiple co-expression modules and identifying genes associated with specific biological processes. In this study, we applied WGCNA method to establish co-expression networks and related them to the results of immune cell infiltration. Through WGCNA analysis, we identified two modules highly correlated with immune cell infiltration and extracted their hub genes: *Lgmn* and *Ntm*. Through a screening of the FerrDb database, we found that *Lgmn* is a FRDEG that regulates oxidation reaction.

The *Lgmn* gene encodes the Legumain protein. The typical trafficking pathway of Legumain is from the endoplasmic reticulum and Golgi apparatus to the lysosome, where it is eventually activated [59]. In addition, Legumain has been found to exist in other locations, such as the cytoplasm, nucleus, and cell surface, indicating it has diverse functions [60]. The extracellular role of Legumain is not fully understood, but it may facilitate communication between different cells and tissues, as secreted Legumain can be internalized and subsequently processed into active forms by various cell types [59, 61]. Furthermore, it has been demonstrated that Legumain is associated with various diseases, including cancer [62], neurodegenerative diseases [63], and so on.

Legumain protein can interact with the ferroptosis inhibitor GPX4 to promote ferroptosis by increasing degradation of GPX4 in lysosomes [26]. In addition, Legumain is involved in immune cell infiltration [64] and can participate in the processing of CD74 and antigen-presenting protein MHCII in B cells [65]. Legumain can also indirectly promote antigen presentation by activating other proteases [66]. In the immune system dependent on TLR7 and/or 9 signaling, Legumain plays multiple roles, participating in the processing and activation of Toll-like receptors in macrophages and dendritic cells, which can recognize microbial molecules and participate in immune response [67–69]. Legumain is highly expressed in the process of monocyte-to-macrophage differentiation [70], and the secretion of Legumain is significantly higher in pro-inflammatory M1 macrophages than in anti-inflammatory M2 macrophages after polarization [71]. However, using recombinant Legumain to treat primary peripheral blood monocytes during differentiation upregulates anti-inflammatory factors and downregulates pro-inflammatory factors, inducing an anti-inflammatory phenotype [72], indicating that Legumain plays a role in regulating immune responses.

## Conclusion

After analyzing the transcriptome data, we found differential expression of genes related to ferroptosis following TBI. In addition, using the WGCNA analysis, we identified modules associated with differentially expressed immune cell genes, where the hub gene *Lgmn* was also identified as a FRDEG. Further analysis revealed that *Lgmn* was highly correlated with multiple ferroptosis markers and immune infiltrating cells. Our results suggest a strong association between immune infiltrating cells and ferroptosis following TBI, and that *Lgmn* may play a significant role in this process.

## Data Availability

Publicly available datasets were analyzed in this study. The datasets GSE128543 for this study can be found here: https://www.ncbi.nlm.nih.gov/geo/.

## Abbreviations

TBI: Traumatic brain injury
GEO: Gene Expression Omnibus
DEGs: Differentially expressed genes
FRDEGs: Ferroptosis-related differentially expressed genes
WGCNA: Weighted gene co-expression network analysis
TOM: Topological overlap matrix
GS: Gene significance
MEs: Module eigengenes
MM: Module membership
GO: Gene ontology
KEGG: Kyoto Encyclopedia of Genes and Genomes
BP: Biological Process
CC: Cellular Component
MF: Molecular Function
ssGSEA: Single-sample Gene Set Enrichment Analysis
PUFAs: Polyunsaturated fatty acids
